# Identifying Four Subgroups of Trauma in Psychosis: Vulnerability, Psychopathology, and Treatment

**DOI:** 10.3389/fpsyt.2017.00021

**Published:** 2017-02-13

**Authors:** Lucy H. Stevens, Helen M. Spencer, Douglas Turkington

**Affiliations:** ^1^Tees Esk and Wear Valley NHS Mental Health Foundation Trust, Cleveland, UK; ^2^Northumberland, Tyne and Wear NHS Mental Health Foundation Trust, Newcastle upon Tyne, UK

**Keywords:** schizophrenia, psychosis, trauma, subgroup, PTSD, CBT

## Introduction and Proposal

Schizophrenia has traditionally been viewed as a unitary diagnostic entity with a single neuropathogenesis (1). However, classification is evolving and schizophrenia is now given less importance in DSM-V (2), with the proposal that schizophrenia spectrum disorders better capture varying manifestations of psychotic symptoms (3). Recent research has pointed to a role for traumatic predisposition in many forms and time points in symptom development. For the first time, four clinical manifestations relating to traumatic vulnerability, trigger, and treatment implications are discussed. Symptoms will be referred to as psychosis, as this has been has been considered an accepted societal term to describe experiences such as hallucinations and delusions (4). However, the focus will remain on symptom presentations seen in schizophrenia spectrum disorders, as the purpose of this piece is to challenge schizophrenia as a unitary diagnostic concept.

## Subgroup 1: Traumatic Psychosis

This first clinical manifestation considers the “classical” association between trauma and psychosis, by referring to childhood trauma leading to an overt traumatic and coexisting schematic vulnerability (5, 6). This increases the risk of the emergence of psychotic symptoms in response to later triggers. In addition to the chronicity and multiplicity of the childhood abuse or neglect experienced (7), other factors have been linked with increased risk, such as perceived intent to harm from the victim’s perspective (8), the closeness of the relationship between victim and perpetrator (9), and whether the victim is older than 12 or 16 years (9, 10). This indicates why childhood abuse, neglect, and bullying can be particularly harmful. Neurobiological research suggests childhood abuse is more likely to affect brain-derived neurotrophic factor expression, leading to dysregulation of the HPA axis and associated dopamine changes (11, 12) giving a putative marker for this subgroup. A meta-analysis supported the existence of traumatic psychosis by suggesting that if childhood abuse was eradicated, then one-third of adult psychosis would not occur (13).

When these individuals present with psychosis, they are more likely to experience positive symptoms (14). This includes a broad spectrum of hallucinatory experiences often congruent with the trauma history (e.g., somatic, olfactory, gustatory, visual, and auditory hallucinations), and also dissociative phenomena and higher levels of depression, anxiety, guilt, and shame (7, 15–17). From clinical observation, secondary substance misuse to self-manage symptoms and severe weight loss or gain as a somatic defense to help prevent further trauma, can occur. Re-traumatization is almost always present, as the trigger for psychosis is either directly congruent with earlier traumatic experiences, or threatens compensatory schema. For example, delusional formation can be protective of schematic beliefs developed in response to trauma (18), whereby life events threaten compensatory schema and lead to the development of delusional mood followed by delusional perception (19). Resulting psychotic symptoms can either be a direct or metaphorical representation of the earlier trauma (20), such as the relationship between paranoid ideas with prominent hypervigilance and formulation congruent themes of punishment and surveillance. Diagnoses can be unstable (i.e., schizoaffective disorder, schizophrenia, borderline personality disorder, dissociative disorders), given the presence of dissociation and self-harm in this subgroup. Markers of this group can be present prior to trauma disclosure. For example, the occurrence of non-epileptic attacks, which typically abate as disclosure proceeds, is a clinical observation that could be considered a dissociative and conversion phenomenon.

Evidence has suggested this could be a subgroup of people who respond poorly to antipsychotic medication (21) but could be a viable target for various psychological treatment options, including expert cognitive behavioral therapy (CBT), which can lead to stabilization and recovery (22). Techniques include building trust, scaffolding of activities, introducing effective coping strategies as well as grounding and stabilization, psychoeducation around dissociation, and techniques for regulating emotions. Crucially, the development of a maintenance formulation linking past trauma with present triggers and threat (23) is a subsequent stage in therapy, as this link has often not been made by the patient. Highlighting the timeline of events and symptoms and gradually working toward insight into emotional hotspots, particularly guilt and shame, can be longer term therapy targets. This enables reliving or restructuring to proceed, at a pace which can be tolerated by the patient, but it must be considered this may never be processed if too painful.

## Subgroup 2: Neurodevelopmental Psychosis

The second subgroup is characterized by those who appear to have a chronic and pervasive vulnerability that is genetic and/or organic in nature. For example, research has suggested a link between the C4 gene and excessive neural pruning during adolescence, which has been linked to the development of psychosis (24). This group could be broadened to consider vulnerabilities posed by neurodevelopmental disorders, but this discussion is beyond the scope of this paper. As children, these individuals may be recognized by teachers, as being socially isolated, displaying clumsiness, and suppressing emotional needs. Persistence of these behaviors can lead to alogia/communication difficulties, poor concentration and memory, affective blunting, poor self-care, and reduced motivation, which traditionally could be interpreted as negative symptoms. Given these difficulties, individuals could be at risk of homelessness, or poor quality accommodation, and hence more vulnerable to victimization. As a result, this can increase vulnerability to post-traumatic stress disorder (PTSD), which may occur as a secondary phenomenon of the trajectory of psychosis (25). Unfortunately, substances can be used to self-medicate symptoms of PTSD in this subgroup. Research has reported increased relative risk of substance abuse in those with PTSD (RR = 4.9), even when compared to people who have experienced trauma but do not have PTSD symptoms (RR = 2.0) (26).

It is important to consider Maslow’s Hierarchy of Needs (27) before commencing treatment as it is crucial to stabilize basic needs, such as sourcing appropriate accommodation and community care, before attempting to address higher level psychological needs. One opinion is that antipsychotic medication can be efficacious for this group and psychosocial interventions such as family therapy, social skills training, and cognitive remediation may be as effective as CBT. The development of graded activities and approaches such as anxiety management, activity scheduling, and social skills training can allow a gentle cognitive restructuring approach to take place over a period of sessions, with variable length and duration. The presence of PTSD in this subgroup can be difficult to detect, but should be enquired about in all cases.

## Subgroup 3: Psychotic PTSD

The third subgroup refers to the symptoms of PTSD developing prior to the onset of psychotic symptoms. This type of psychosis, similar to the traumatic psychosis subgroup, is triggered by trauma, but the symptoms may be a response to an initial trauma of any nature (rather than re-traumatization) and the symptoms of PTSD predate the emergence of the symptoms of psychosis (28). For example, individuals in the traumatic psychosis subgroup may develop a voice that resembles a flashback in an auditory modality, but the initial presentation in this subgroup is more like typical PTSD symptoms. Flashbacks and nightmares, resulting in insomnia and increased arousal, are followed by psychotic symptoms overtly linked to the trauma. For example, vulnerability to developing psychosis in this subgroup (e.g., perceptual phenomena) can be a result of prolonged exposure to threatening stimuli and extreme hypervigilance, perhaps *via* sleep deprivation associated with these symptoms.

Individuals in this subgroup would be expected to respond to expert CBT directly targeting the PTSD. For example, use of the cognitive model of PTSD (23) is crucial for introducing the link between past and current experiences and hence creating a rationale for trauma-focused intervention as well as quickly establishing triggers and maintenance factors in the current environment. In line with evidence-based practice for PTSD (29), both trauma-focused CBT (TF-CBT) and eye movement desensitization and reprocessing (EMDR) are viable and involve work on emotional hotspots (such as shame and anger) along with cognitive restructuring or imaginal reliving.

## Subgroup 4: Psychosis-Induced PTSD

The final subgroup relates to the triggering of PTSD as a result of acute psychosis (30). This could be owing to loss of insight, leading to high levels of perceived threat and conviction in distressing delusional beliefs. It could also relate to previous traumas that have been suppressed, where re-traumatization occurs. For example, a person on an inpatient ward who tried to abscond believing that the nursing staff were planning to castrate him, resulted in a nurse grabbing hold of him and pulling down his trousers for the purposes of rapid tranquilization. This acted to confirm his beliefs that he was going to be castrated. If the same person had experienced sexual trauma or abuse, this could result in re-traumatization through activation of existing beliefs. Both might have resulted in PTSD. Although re-traumatization is possible in this subgroup, this is different to the triggers described in the traumatic psychosis subgroup, as the crucial trigger here is the episode of psychosis, which predates PTSD symptoms. It is crucial that PTSD is detected and managed, but quite often this is not the case and a lack of understanding can act as a powerful maintenance factor for chronicity and relapse to occur. For example, individuals may become mistrustful of their own self-awareness and ability to cope, often owing to a later acknowledgment that they themselves had no insight into their own deterioration of mental state during the initial psychotic episode. This can be a powerful maintaining factor in subsequent episodes owing to high threat sensitivity for perceptual/psychotic phenomena.

In addition to TF-CBT for PTSD, there also needs to be a strong emphasis on making sense of, and understanding, the experiences of psychosis in the context of the person’s life. Some psychoeducation around hypervigilance would also be important, in the context of monitoring bodily experiences and interpreting these in a particular way (e.g., as in health anxiety). This should help to inform a robust relapse plan that emphasizes a balance between recognizing signs of relapse, but also being aware of hypervigilance to threat and the impact that this can have on misinterpretation of bodily experiences.

## Summary and Future Directions

Four subgroups of traumatic psychosis have been briefly described in relation to mechanisms of action and recommended intervention modalities, based on clinical practice and the available literature. This paper supports the argument that schizophrenia should be explored in a symptom-specific way, given the varying symptom presentations and multifactorial means of understanding the trajectories of trauma and psychosis. It is clear that this is more complex than the initial conceptualization of traumatic psychosis. This paper intended to provide a summary, and hence further research is needed to understand other clinical presentations. Although this paper discussed subgroups in the context of schizophrenia spectrum disorders, it is not implied that these presentations are limited to such, given the presentation of psychosis across diagnostic categories. Also, it is not always intuitive or possible to consider these subgroups as discrete categories. However, the nature and timing of the trauma, and initial symptom presentation can be a means of identifying these subgroups and guiding treatment.

By developing further subdivisions of psychosis based on potential mechanisms of trigger, these categories are formulation driven, which is beneficial for informing evidence-based interventions, as demonstrated in this paper. There are many pioneering approaches beyond the scope of this paper that may provide efficacy in managing trauma and perceptual phenomena, which should be considered further. These include EMDR, method of levels, acceptance and commitment therapy, compassion-focused therapy, dialectical behavioral therapy, and imagery reprocessing.

## Author Contributions

We confirm that all authors contributed equally to the writing of this manuscript.

## Conflict of Interest Statement

LS and DT are practitioners of cognitive behavioral therapy (CBT) and deliver this intervention within the UK National Health Service. DT receives royalties from texts or books published on cognitive therapy. HS and DT have received fees for delivering workshops on cognitive therapy. DT has received lecture fees from pharmaceutical companies.
